# Resistance exercise improves physical fatigue in women with fibromyalgia: a randomized controlled trial

**DOI:** 10.1186/s13075-016-1073-3

**Published:** 2016-07-30

**Authors:** Anna Ericsson, Annie Palstam, Anette Larsson, Monika Löfgren, Indre Bileviciute-Ljungar, Jan Bjersing, Björn Gerdle, Eva Kosek, Kaisa Mannerkorpi

**Affiliations:** 1 Institute of Neuroscience and Physiology/Physiotherapy, Sahlgrenska Academy, University of Gothenburg, Göteborg, Sweden; 2 University of Gothenburg Centre for Person Centered Care (GPCC), Göteborg, Sweden; 3 Karolinska Institutet, Department of Clinical Sciences, Danderyd Hospital Stockholm, Stockholm, Sweden; 4 Institute of Medicine, Department of Rheumatology and Inflammation research, Sahlgrenska Academy, University of Gothenburg, Göteborg, Sweden; 5 Department of Medical and Health Sciences, Division of Community Medicine, Faculty of Medicine and Health Sciences, Linköping University, Pain and Rehabilitation Center, Anesthetics, Operations and Specialty Surgery Center, Region Östergötland, Linköping, Sweden; 6 Department of Clinical Neuroscience, Karolinska Institutet, Stockholm, Sweden

**Keywords:** Fibromyalgia, Resistance exercise, Exercise, Fatigue, Randomized controlled trial

## Abstract

**Background:**

Fibromyalgia (FM) affects approximately 1–3 % of the general population. Fatigue limits the work ability and social life of patients with FM. A few studies of physical exercise have included measures of fatigue in FM, indicating that exercise can decrease fatigue levels. There is limited knowledge about the effects of resistance exercise on multiple dimensions of fatigue in FM. The present study is a sub-study of a multicenter randomized controlled trial in women with FM. The purpose of the present sub-study was to examine the effects of a person-centered progressive resistance exercise program on multiple dimensions of fatigue in women with FM, and to investigate predictors of the potential change in fatigue.

**Methods:**

A total of 130 women with FM (age 22–64 years) were included in this assessor-blinded randomized controlled multicenter trial examining the effects of person-centered progressive resistance exercise compared with an active control group. The intervention was performed twice a week for 15 weeks. Outcomes were five dimensions of fatigue measured with the Multidimensional Fatigue Inventory (MFI-20). Information about background was collected and the women also completed several health-related questionnaires. Multiple linear stepwise regression was used to analyze predictors of change in fatigue in the total population.

**Results:**

A higher improvement was found at the post-treatment examination for change in the resistance exercise group, as compared to change in the active control group in the MFI-20 subscale of physical fatigue (resistance group Δ –1.7, SD 4.3, controls Δ 0.0, SD 2.7, *p* = 0.013), with an effect size of 0.33. Sleep efficiency was the strongest predictor of change in the MFI-20 subscale general fatigue (beta = −0.54, *p* = 0.031, *R*^2^ = 0.05). Participating in resistance exercise (beta = 1.90, *p* = 0.010) and working fewer hours per week (beta = 0.84, *p* = 0.005) were independent significant predictors of change in physical fatigue (*R*^2^ = 0.14).

**Conclusions:**

Person-centered progressive resistance exercise improved physical fatigue in women with FM when compared to an active control group.

**Trial registration:**

ClinicalTrials.gov NCT01226784. Registered 21 October 2010.

## Background

The prevalence of fibromyalgia (FM) is approximately 1–3 % in the general population, increases with age, and is more prevalent in women than in men [1]. In addition to persistent widespread pain and allodynia, patients with FM often experience severe fatigue, sleep disturbances, stiffness, psychological distress and/or cognitive impairment [2–5]. Women with FM have been shown to have impaired physical capacity [6–8] and they are also less physically active compared to healthy controls [9]. FM is a dominating cause of sick leave among female patients with musculoskeletal disorders and has a great impact on quality of life [6, 10].

Fatigue is a prominent symptom in patients with FM, which limits their work ability and social life [11–13]. Women with FM have described their fatigue in terms of sleepless nights, physical weakness, social withdrawal, loss of mental energy and overwhelming exhaustion [13]. Socio-demographic aspects such as female gender, younger age, low working capacity and low level of education have been shown to be associated with higher levels of fatigue in FM [14].

Fatigue is commonly assessed with a one-dimensional visual analog scale (VAS), which enables comparisons across studies. However, fatigue is a complex symptom that interacts with several other common FM symptoms. It is thus recommended to be assessed in multiple dimensions, such as physical, mental and general fatigue in patients with FM, especially in non-pharmacological studies [15, 16]. Fatigue has been shown to be associated with increased distress, muscular tenderness, and poor sleep quality [14, 15, 17, 18]. Higher ratings on the subscales of physical fatigue and reduced activity, which are included in the Multidimensional Fatigue Inventory (MFI-20), have been associated with low working capacity, low level of physical activity and impaired physical capacity in women with FM [15, 19, 20].

Pain catastrophizing influences the perception of pain and other symptoms in many patients with FM, which complicates the treatment and could influence compliance with exercise [21]. Catastrophic thinking has also been suggested to have an influence on fatigue in FM and in other chronic conditions. However previous research on this topic is scarce [22].

The most beneficial treatment for FM requires a multidisciplinary approach combining education, pharmacological treatment, exercise and cognitive behavioral therapy [23, 24]. Exercise has been found to improve feelings of energy and fatigue in various medical conditions [25]. Different types of exercise are being used for treatment in FM and chronic widespread pain (CWP) in health care, such as aerobic exercise, resistance training, flexibility exercise and body awareness therapy [26].

Few exercise studies have included measures of fatigue in patients with FM. However the findings of these studies indicate that exercise could decrease fatigue levels [26–29]. Aerobic exercise has been shown to improve the MFI-20 dimension of reduced motivation in female patients with FM [30, 31] and global outcome measures of physical capacity and, to some degree, pain and the number of tender points in FM [26].

Resistance exercise has shown positive effects on limitations in activity, pain, global fatigue, depression and muscle strength in patients with FM. However, the quality of evidence of these effects is poor due to the limited number of studies [29]. The present study is a sub-study of a randomized controlled trial (RCT) showing that resistance exercise improved muscle strength, overall health, and current pain intensity in women with FM, when compared to an active control group [32]. The resistance exercise intervention was a person-centered approach, which emphasizes active involvement of the patient in planning the treatment that is suggested to enhance the patient’s ability to manage health problems [33]. The principles of person-centeredness were used in the previously published RCT. The details of the exercise program were planned together with each patient to support each participant’s ability to manage the exercise and the progression in loads [32].

As high levels of fatigue have been associated with low levels of physical activity and impaired physical capacity in FM [15, 19, 20], improvement in physical capacity in patients with FM may result in a reduction in fatigue. To our best knowledge, there is no previous study investigating the effects of resistance exercise on multiple dimensions of fatigue in FM.

The primary aim of the present study was to examine the effects of a person-centered progressive resistance exercise program on multiple dimensions of fatigue in women with FM compared to an active control group, and to investigate predictors of the potential change in fatigue. Second, the effect of resistance exercise on sleep, pain catastrophizing, depression, and anxiety were also explored, as these variables have been shown previously to be associated with fatigue.

## Methods

### Study design

This is a sub-study of a multicenter randomized controlled trial in women with FM (ClinicalTrials.gov identification number: NCT01226784) [32]. The present sub-study aimed to investigate the effects of resistance exercise on multidimensional fatigue in women with FM.

### Recruitment

Inclusion and exclusion criteria have been described in detail previously [32]. In short, the inclusion criteria were women aged 20–65 years, meeting the American College of Rheumatology (ACR) 1990 classification criteria for FM [2], and the exclusion criteria were other severe somatic or psychiatric disorders, participation in a rehabilitation program within the past year, or inability to understand Swedish.

**Fig. 1:**
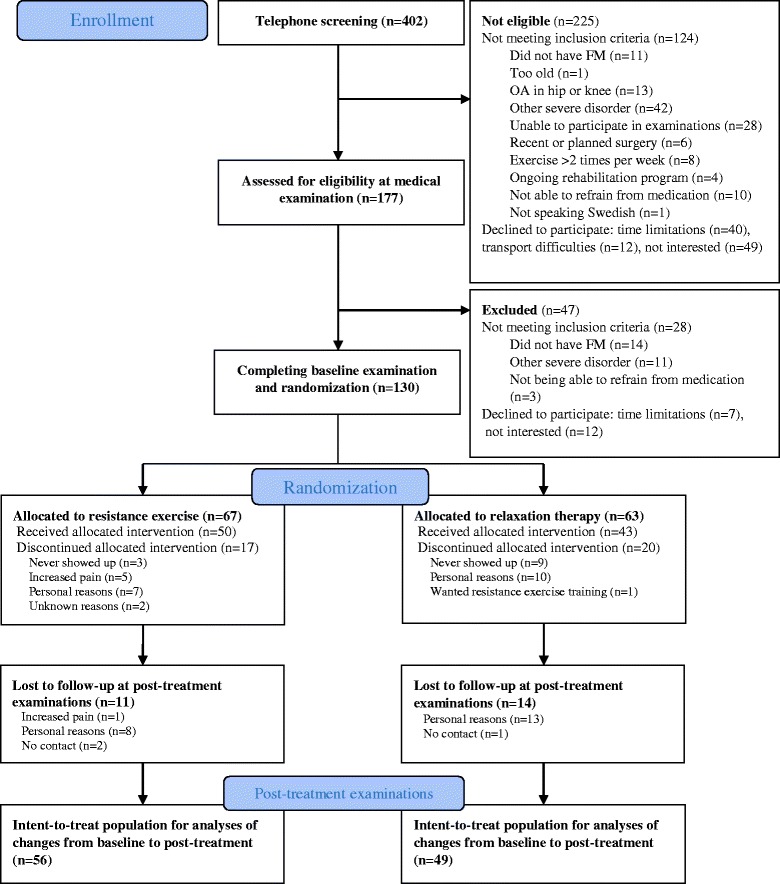
Consolidated Standards of Reporting Trials (CONSORT) flow diagram of the progress of the two groups in the randomized trial. Modified figure from [32]. *FM* fibromyalgia, OA osteoarthritis

Female patients with FM were recruited by newspaper advertisement in the local newspapers of three cities in Sweden (Gothenburg, Stockholm, and Linköping) to the multicenter experimental study [32]. There were 130 patients with FM included in the study, and they were randomized to the resistance exercise group (*n* = 67) or the active control group (*n* = 63). The process of recruitment and randomization has been described in detail in a previous publication [32]. No significant differences were found in sociodemographic data between the resistance exercise group (*n* = 67) and the active control group (*n* = 63) (Fig. 1) [32].

All participants were invited to a post-treatment examination after 15 weeks and 84 % (*n* = 56) in the resistance exercise group and 76 % (*n* = 49) in the active control group completed the test. Seventeen participants (25 %) in the resistance exercise group and 20 (32 %) in the active control group discontinued the intervention due to various reasons [32]. Five participants in the resistance exercise group reported adverse effects and chose to discontinue the intervention due to increased pain. Two of these participants completed post-treatment examinations (Fig. 1). Modified figure from [32].

### Interventions

#### Resistance exercise

The person-centered progressive resistance exercise intervention was performed twice a week for 15 weeks at physiotherapy premises and at a local gym and was supervised by experienced physiotherapists. The exercise program was standardized and performed in groups of five to seven participants but the load was adjusted individually. The exercise session started with 10 minutes of warm up followed by 50 minutes of resistance exercises focused on large muscle groups in all four extremities and trunk. The resistance exercise was initiated at 40 % of 1 repetition maximum (RM) and progressed up to 80 % of 1 RM during the 15 weeks. Possibilities for progression of loads were evaluated every 3–4 weeks. Forty-two participants (62.7 %) in the resistance exercise group reached exercise loads of 80 % of 1 RM while seven participants (10.4 %) reached exercise loads of 60 % of 1 RM. The median attendance rate at the resistance exercise sessions was 71 % (range 0–100 %). The procedure of the resistance exercise and adjustment of loads in the present study has been described in detail previously [32].

#### Active control group

The active control treatment consisted of relaxation therapy, which was performed twice a week for 15 weeks, guided by experienced physiotherapists and conducted at physiotherapy premises in groups of five to eight participants. It was performed as autogenic training [34], which refers to a series of mental exercises including autosuggestion and relaxation. The relaxation therapy lasted for approximately 25 minutes, followed by stretching exercises. The median attendance rate at the relaxation therapy sessions was 64 % (range 0–100 %). The procedure in the active control group has been described in detail previously [32].

### Measurements

The patients were referred to a baseline examination which included a tender-point examination [2] by a physician, performance-based tests of physical capacity conducted by physical therapists, and a battery of self-reported questionnaires [32].

### Background data

Background data such as age, education, employment, duration of pain, sick leave, disability pension, and medication were obtained by a standardized interview. Duration of widespread pain was reported in years and obtained by a standardized interview. Employment was divided into four categories of percentage of full-time work: 0 %; 1–49 %; 50–79 %; and 80–100 %. Full-time work was defined as 40 h per week.

### Self-reported questionnaires

#### Multidimensional Fatigue Inventory *(4–20)*

The MFI-20 comprises 20 statements on a 5-point Likert scale that addresses aspects of fatigue experienced during the most recent days. The scores generate five subscales of fatigue: general fatigue, physical fatigue, mental fatigue, reduced motivation, and reduced activity. The scores range from 4 to 20 for each subscale and higher scores refer to a higher degree of fatigue [35, 36]. The MFI-20 has been shown to have satisfactory convergent construct validity and test-retest reliability in FM [37].

#### FIQ fatigue *(0–100)*

The VAS for fatigue included in the Fibromyalgia Impact Questionnaire (FIQ) [38] was used as a one-dimensional measure of fatigue in this study, ranging from 0 mm (no tiredness) to 100 mm (very tired). The FIQ for fatigue has been validated and shown satisfactory test-retest reliability in Swedish patients with FM [39].

#### FIQ pain *(0–100)*

The VAS for pain is a subscale of the FIQ that measures current pain intensity. The participant was asked to rate her current pain intensity, ranging from 0 mm (no pain at all) to 100 mm (worst imaginable pain) [38]. The FIQ for pain was used only in the analyses of predictors of potential change in fatigue.

#### Pittsburgh Sleep Quality Index (PSQI) *(0–21)*

The PSQI assesses sleep quality and disturbances over a 1-month period. It comprises nineteen items (scored 0–3), which constitute seven subscales: sleep quality, sleep latency, sleep duration, sleep efficiency, sleep disturbances, need of medications to sleep (use of sleeping medication) and daytime dysfunction. The sum of scores for the seven subscales generate one total score (0–21). A PSQI total score >5 indicates poor sleep [40]. The PSQI has been used previously in studies of FM [41, 42].

#### Pain catastrophizing scale (PCS) *(0–52)*

The PCS assesses pain-related catastrophic thinking. The patients estimate on a 5-point Likert scale (0 (not at all) to 4 (all the time)) the extent to which they experience 13 thoughts or feelings while they feel pain. The PCS total score (0–52) is the sum of the scores of the 13 items [43]. A PCS total score >24 displays a high level of catastrophic thinking in patients with sub-acute whiplash-related pain [44]. The 13 items also generate three subscales; rumination, helplessness and magnification [43]. The three PCS subscales and the total score were used in the present study in between-group analyses of change over time, while only the PCS total score was used in the analyses of predictors of change in fatigue. The PCS has been tested for validity and reliability [45, 46] and has been used in studies of FM [47–49]

#### Hospital Anxiety and Depression Scale (HADS) *(0–21)*

The HADS contains 14 statements, ranging from 0–3, and a higher score refers to a higher degree of distress. The scores of the 14 items build two subscales: HADS-A for anxiety (0–21) and HADS-D for depression (0–21) [50]. A cutoff score of 8 is suggested to indicate possible anxiety or depression [51].

### Tests of physical capacity

#### Leisure Time Physical Activity Instrument (LTPAI) *(h)*

The patients estimate their amount of physical activity in leisure time during a typical week [52]. The physical activities were divided into three categories: light, moderate, and heavy activities, and examples of such activities were given for each category. The LTPAI total score is the sum of hours in all three categories and was used in the present study only in the analyses of potential predictors of change in fatigue.

#### The 6-minute walk test (6MWT) *(m)*

The patient was instructed to walk for 6 minutes as quickly as she could without running and the total walking distance was measured [53]. The 6MWT was used in the present study only in the analyses of potential predictors of change in fatigue.

### Statistics

Data were computerized and analyzed using the Statistical Package Software for the Social Sciences (SPSS version 22, Chicago, IL, USA). Descriptive statistics are presented as mean, standard deviation (SD) and range for continuous variables and as number (*n*) and percent (%) for categorical variables.

For comparison between two groups, normal distribution of data collected with questionnaires could not be assumed and the non-parametric Mann–Whitney *U* test was used for continuous variables, Mantel Haenszel chi-squared test for ordered categorical variables and Fisher’s exact test for dichotomous variables. Change over time from baseline to 15 weeks within groups was analyzed using the Wilcoxon signed rank test. Effect size was calculated with Cohen’s *d* for outcomes showing a significant change, by dividing the mean difference between the post-treatment score and baseline score in the resistance exercise group and in the active control group by the pooled SD for difference. Effect sizes from 0.20 to <0.50 were regarded as small and effect sizes from 0.50 to <0.80 were regarded as moderate [54].

A baseline difference was found between the groups in the PSQI subscale of sleep efficiency. The between-group analyses were therefore adjusted for PSQI sleep efficiency in linear regression analyses with outcome variables (mean difference post treatment compared to baseline) included as dependent variables and the group variable (intervention/active control) and PSQI sleep efficiency included as independent variables. The assumptions of normality in regression analyses were confirmed by checking the residual scatter plots and histograms of each variable, respectively. To control for possible type 1 errors, the upper limit of the expected number of false significant results for analyses of primary outcomes (the five subscales of the MFI-20) was calculated by the following formula:

$$ \upalpha /1\ \hbox{--}\ \upalpha \times \left(\mathrm{Number}\ \mathrm{of}\ \mathrm{tests}\ \hbox{--}\ \mathrm{Number}\ \mathrm{of}\ \mathrm{significant}\ \mathrm{tests}\right), $$

where α is the significance level.

Correlation was assessed between the difference between post-treatment and baseline in the five subscales of the MFI-20 and the baseline values for the variables age, employment, duration of widespread pain, FIQ pain, the PCS, the HADS, the PSQI, the LTPAI and the 6MWT, using Spearman’s correlation coefficient (*r*_s_). These variables were chosen based on results from previous studies of correlation between fatigue and other patient characteristics [15, 22]. In order to find independent baseline predictors of change in fatigue for each MFI-20 subscale, the variables with a Spearman’s correlation *p* value *p* <0.1 with the MFI-20 subscale and group of randomization were entered into a multiple linear stepwise regression analysis with change in the fatigue subscale as the dependent variable. Multiple linear stepwise regression analyses were performed of the subscales of the MFI-20 that were found to be significantly correlated with more than one variable. All tests were two-sided and conducted at the 5 % significance level.

## Results

### Between-group comparisons

Results of between-group comparisons are shown in Table 1.

**Table 1:** Baseline values, change from baseline in outcome variables, and within-group and between-group differences in change in the resistance exercise group and the active control group

	Resistance exercise group			Active control group			Resistance vs active control group		
	Baseline *n* = 67 Mean (SD)	∆ 15 weeks *n* = 56 Mean (SD), min, max	Within-group analysis *p* value	Baseline *n* = 63 Mean (SD)	∆ 15 weeks *n* = 49 Mean (SD), min, max	Within-group analysis *p* value	Between-group analysis of baseline *p* value	Between group analysis of change *p* value	Between-group analysis of change, adjusted *p* value^*^
MFI-20									
General fatigue	17.3 (2.7)	−1.3 (3.1), −10, – 7	**0.003**	17.8 (2.74)	−0.5 (2.6), −9, – 4	0.48	0.17	**0.031**	0.37
Physical fatigue	16.0 (3.0)	−1.7 (4.3), −13, – 8	**0.011**	16.5 (2.9)	0.0 (2.7), −8, – 5	0.66	0.29	**0.013**	**0.044**
Mental fatigue	15.0 (3.3)	−1.6 (3.4), −10, – 7	**0.001**	15.0 (4.0)	−0.1 (2.5), −6, – 5	0.85	0.56	**0.008**	0.070
Reduced activity	14.6 (3.4)	−1.0 (3.6), −13, – 6	0.055	15.2 (3.5)	−0.1 (2.8), −8, – 5	0.89	0.29	0.12	0.069
Reduced motivation	10.5 (3.5)	−0.6 (3.7), −10, – 10	0.16	10.4 (3.6)	0.4 (2.8), −8, – 6	0.34	0.78	0.061	0.32
*Exploratory outcomes*									
FIQ fatigue	81.4 (17.3)	−8.6 (21.2), −87, – 66	**0.002**	81.8 (15.8)	−5.5 (19.0), −81, – 73	0.11	0.95	0.27	0.18
PSQI									
Duration of sleep	1.1 (1.1)	−0.0 (0.9), −3, – 3	0.73	1.1 (1.2)	−0.2 (0.9), −2, – 2	0.22	0.94	0.46	0.26
Sleep efficiency	1.9 (1.1)	−0.3 (1.0), −3, – 1	0.061	1.4 (1.2)	0.1 (1.2), −2, – 3	0.55	**0.027**	0.14	0.37
Sleep disturbance	2.0 (0.6)	0.0 (0.6), −1, – 1	1.00	2.0 (0.6)	0.1 (0.6), −1, – 1	0.35	0.89	0.50	0.37
Sleep latency	1.7 (1.0)	−0.1 (0.8), −2, – 1	0.26	1.8 (1.0)	0.0 (0.8), −2,– 2	0.84	0.83	0.53	0.32
Day dysfunction	1.6 (0.8)	−0.1 (0.8), −2, – 2	0.45	1.7 (0.8)	0.0 (0.8), −2, – 2	1.00	0.49	0.64	0.65
Sleep quality	1.8 (0.8)	−0.2 (0.8), −2, – 1	**0.047**	1.9 (0.8)	0.0 (0.8), −1, – 2	0.69	0.90	0.19	0.16
Need meds to sleep	1.0 (1.3)	−0.1 (1.2), −3, – 3	0.69	1.0 (1.3)	0.3 (1.0), −2, – 3	**0.036**	0.82	0.27	0.10
PSQI total score	10.9 (4.3)	−0.6 (3.4), −11, – 9	0.18	10.8 (4.0)	0.5 (3.0), −5, – 9	0.48	0.76	0.13	0.23
PCS									
Rumination	6.3 (3.6)	−0.9 (3.5), −8, – 12	**0.046**	6.6 (4.3)	−0.8 (3.2), −8, – 10	0.056	0.81	0.97	0.11
Magnification	3.4 (2.5)	−0.6 (1.9), − 4, – 4	**0.030**	3.6 (2.8)	−0.4 (2.0), −5, – 3	0.25	0.82	0.50	0.18
Helplessness	9.7 (5.3)	−1.3 (4.0), −10, – 9	**0.012**	10.1 (5.9)	−1.6 (4.6), −15, – 6	0.051	0.84	0.84	0.49
PCS total score	19.4 (10.0)	−2.7 (7.6), −17, – 19	**0.004**	20.3 (11.9)	−2.8 (7.9), −25, – 11	0.055	0.88	0.58	0.16
HADS									
Depression	7.0 (3.9)	−0.7 (3.7), −9, – 11	0.086	6.7 (3.5)	0.3 (2.8), −6, – 12	0.49	0.80	0.082	0.11
Anxiety	7.9 (4.7)	−0.3 (3.6), − 11, – 8	0.58	8.0 (4.5)	0.5 (2.7), − 9, – 8	0.10	0.94	0.22	0.099

*MFI-20* Multidimensional Fatigue Inventory, *FIQ* Fibromyalgia Impact Questionnaire, *PSQI* Pittsburgh Sleep Quality Index, *PCS* Pain Catastrophizing Scale, *HADS* Hospital Anxiety and Depression Scale

^*^ *p* value adjusted for PSQI sleep efficiency

*p* values <0.05 are marked in bold print

#### Multidimensional fatigue

There was significantly greater improvement at the post-treatment examination according to change in the MFI-20 subscales for general fatigue (*p* = 0.031), physical fatigue (*p* = 0.013) and mental fatigue (*p* = 0.008) in the resistance exercise group, as compared to the change over time in the active control group (Table 1). The mean improvements in the resistance exercise group from baseline to post treatment were 7.5 % in general fatigue, 10.6 % in MFI-20 physical fatigue and 10.7 % in mental fatigue. There were no statistically significant differences between the two groups in change in the MFI-20 subscales for reduced motivation or reduced activity at the post-treatment examination (Table 1).

There was a baseline difference between the resistance exercise group and the active control group in the PSQI subscale of sleep efficiency (mean 1.9, SD 1.1 versus mean 1.4, SD 1.2, *p* = 0.027) (Table 1). When the between-group analyses of the MFI-20 were adjusted for PSQI sleep efficiency, the difference between groups in change of fatigue was significant only for the MFI-20 subscale physical fatigue (*p* = 0.044) (Table 1). The effect size of change in MFI-20 for physical fatigue in the resistance exercise group compared to the active control group was 0.33 (i.e., a small effect size).

Type 1 error: the between-group analyses of primary outcomes (the five subscales of the MFI-20) comprised a total of five statistical analyses, with one significant value at the significance level 0.05, and the upper level of the number of false significance results was 0.21, which indicates that 0–1 of the significant results observed might be false.

#### Exploratory outcomes

No statistically significant differences were found between the resistance exercise group and the relaxation group in change in the FIQ for fatigue, the PSQI, the PCS or the HADS at the post-treatment examination (Table 1).

### Within-group analyses of exploratory outcomes, and change from baseline to post treatment

Results of within-group analyses of outcomes, and change from baseline to post treatment are shown in Table 1.

#### The FIQ for fatigue

The resistance exercise group improved in the FIQ for fatigue over time from baseline to post treatment (mean difference −8.6, SD 21.2, *p* = 0.002) (Table 1).

#### The PSQI

The resistance exercise group improved over time in the PSQI subscale for sleep quality (mean difference −0.2, SD 0.8, *p* = 0.047), while the active control group improved in the PSQI subscale for need of medications to sleep (mean difference 0.3 SD 1.0, *p* = 0.036) (Table 1).

#### The PCS

The resistance exercise group improved significantly over time in all three PCS subscales and the PCS total score (mean difference in PCS total score −2.7 SD 7.6, *p* = 0.004). In the active control group there was a tendency towards improvement in two PCS subscales and the PCS total score (*p* = 0.051–0.056) (Table 1).

#### The HADS

No significant changes during the study period were found within any of the groups for HADS anxiety or HADS depression (Table 1).

### Predictors of change in fatigue

The baseline values of the variables included in the correlation analysis are presented in Table 1 and in a previous publication [32]. The results of the correlation analysis are presented in Table 2. Multiple linear stepwise regression analyses were carried out on the subscales of the MFI-20 that were found to be significantly correlated with more than one variable, which were general fatigue and physical fatigue. Variables correlated with change in MFI-20 subscales with a *p* value <0.1 were included together with intervention group (resistance/control) in the multiple linear stepwise regression analyses of predictors of change in the MFI-20 subscales.

**Table 2:** Spearman’s correlation coefficients (*r*_s_) with *p* values for correlation between change (posttest-baseline) in the five subscales of the Multidimensional Fatigue Inventory-20 and patient characteristics, symptoms and physical function (*n* = 105)

	Change in general fatigue *r*_s_, *p* value	Change in physical fatigue *r*_s_, *p* value	Change in mental fatigue *r*_s_, *p* value	Change in reduced activity *r*_s_, *p* value	Change in reduced motivation *r*_s_, *p* value
Age	−0.09, 0.36	0.00, 0.98	0.01, 0.89	0.027, 0.78	0.033, 0.74
Employment	**0.23, 0.021**	**0.26, 0.008**	0.09, 0.35	0.11, 0.28	0.05, 0.62
Duration CWP	0.00, 0.98	−0.07, 0.47	0.04, 0.70	0.10, 0.33	0.10, 0.33
FIQ pain	**−0.17, 0.079**	−0.14, 0.15	0.15, 0.12	−0.12, 0.23	0.02, 0.85
PCS total	0.02, 0.84	0.03, 0.80	0.02, 0.86	−0.04, 0.69	0.09, 0.39
HADS-D	0.00, 0.98	−0.02, 0.83	−0.12, 0.21	0.04, 0.70	−0.030, 0.76
HADS-A	0.06, 0.52	0.11, 0.26	−0.11, 0.28	0.14, 0.16	0.12, 0.24
PSQI					
*Duration of sleep*	−0.02, 0.84	−0.06, 0.55	−0.09, 0.39	−0.06, 0.58	−0.10, 0.36
*Sleep efficiency*	**−0.21, 0.049**	**−0.21, 0.044**	**−0.21, 0.048**	−0.06, 0.54	**−0.22, 0.037**
*Sleep disturbance*	−0.03, 0.74	0.09, 0.39	0.04, 0.67	0.16, 0.12	0.04, 0.69
*Sleep latency*	−0.15, 0.15	−0.05, 0.62	−0.05, 0.66	−0.08, 0.46	−0.07, 0.54
*Day dysfunction*	0.06, 0.52	0.03, 0.74	0.00, 0.95	−0.08, 0.40	0.14, 0.18
*Sleep quality*	−0.03, 0.75	−0.02, 0.84	−0.05, 0.63	0.05, 0.63	0.14, 0.17
*Need meds to sleep*	−0.14, 0.15	−0.08, 0.41	−0.11, 0.28	0.00, 0.99	0.03, 0.80
*Total score*	−0.10, 0.31	−0.11, 0.30	−0.13, 0.20	−0.06, 0.53	−0.4, 0.67
LTPAI	−0.09, 0.38	0.08, 0.44	−0.02, 0.83	0.11, 0.29	−0.04, 0.69
6-minute walk test	0.06, 0.53	0.10, 0.33	−0.09, 0.35	−0.04, 0.71	−0.13, 0.19

Correlation coefficients with a *p* value <0.1 are marked in bold print. *CWP* chronic widespread pain, *FIQ* Fibromyalgia Impact Questionnaire, *PCS* Pain Catastrophizing Scale, *HADS-D* Hospital Anxiety and Depression Scale – subscale of depression, *HADS-A* Hospital Anxiety and Depression Scale – subscale of anxiety, *PSQI* Pittsburgh Sleep Quality Index, *meds* medications, *LTPAI* Leisure Time Physical Activity Instrument

#### MFI-20 general fatigue

Intervention (resistance/control), employment, the PSQI for sleep efficiency and the FIQ for pain were associated (*p* < 0.1) with change in the MFI-20 for general fatigue (Tables 1 and 2) and were included in the multiple linear stepwise regression analysis for general fatigue. After the PSQI for sleep efficiency was entered into the stepwise model as the first variable, no additional variable was entered (*B* = − 0.54, SE 0.25, *p* = 0.031). The *R*^2^ value for the model was 0.05. Poorer sleep efficiency at baseline predicted improvement in general fatigue.

#### MFI-20 physical fatigue

Intervention (resistance/control), employment and the PSQI for sleep efficiency were associated (*p* < 0.1) with change in the MFI-20 for physical fatigue (Tables 1, 2) and were included in multiple linear stepwise regression analysis of physical fatigue. Intervention (*B* = 1.90, SE = 0.73, *p* = 0.010) and employment status (*B* = 0.8, SE = 0.29, *p* = 0.005) were independent predictors of change in physical fatigue; *R*^2^ for the model was 0.14. Participating in the resistance exercise intervention and working fewer hours per week at baseline predicted improvement in physical fatigue.

## Discussion

The present randomized controlled trial investigated the effects of a 15-week person-centered progressive resistance exercise program on multiple dimensions of fatigue in 130 women with FM. Significant improvements were found for change in the MFI-20 subscales for general fatigue, physical fatigue, and mental fatigue in the resistance exercise group in comparison with the active control group. When the analyses were adjusted for baseline differences in sleep efficiency the between-group difference was significant for MFI-20 physical fatigue only. The effect size for MFI-20 physical fatigue was small (0.33) but lies within the expected range according to a recent meta-analysis, which investigated the effects of exercise on fatigue in patients with FM [55]. The MFI-20 physical subscale reflects fatigue related to “physical ability to do things” and “physical condition”, physical components that are expected to improve with exercise. Although the improvement in physical fatigue was small in effect size, it is valuable for patients describing themselves as physically weak and becoming fatigued after doing very little [56], which causes deterioration in their quality of life and ability to manage daily activities. The MFI-20 subscale for physical fatigue has been used separately by the Outcome Measures in Rheumatology (OMERACT) group in analyses aiming to identify subgroups in FM [57].

A previous study found that decreased fatigue in patients with FM engaging in exercise was associated with changes in adipokines and insulin-like growth factor-1, which appear to be biological correlates of exercise and fatigue [58]. We were also interested in investigating which variables could predict the possible change in fatigue. Participating in resistance exercise combined with working fewer hours per week at baseline predicted greater improvement in the MFI-20 subscale for physical fatigue. The PSQI for sleep efficiency predicted improvement in the MFI-20 for general fatigue, which indicates that the women with FM who had worse sleep efficiency at baseline were more likely to improve their general fatigue regardless of whether they participated in resistance exercise or in the active control group. Sleep efficiency in the PSQI refers to the time asleep per night in relation to the time spent in bed. These findings indicate that women with FM who have the worst sleep efficiency might have the most to gain from any intervention. However, the explained variance ranged from 5 –4 % in the analyses of the MFI-20 subscales, and the improvement in fatigue was reasonably influenced by other factors not investigated in the present study.

Variables that have been previously found to be associated with dimensions of fatigue were included in the correlation analyses; however, only a few variables appeared to be associated with change over time in fatigue. The participants’ age or duration of pain did not appear to have an influence on change in fatigue, nor did their level of psychological distress or physical capacity. These results indicate that women with FM could gain improvements in fatigue by resistance exercise regardless of individual factors. Similar results have also been found for improvements in pain disability [59].

Fatigue is a symptom with a great negative effect on daily life in women with FM [11, 13, 60] and has been recommended to be assessed in multiple dimensions [15, 61], yet the effects of resistance exercise on multidimensional fatigue in FM have not been previously studied. However, a few studies have investigated the effect of resistance exercise on global fatigue assessed with a VAS [62, 63]. In the present study the FIQ for global fatigue was included for exploratory analysis. There was significant improvement in the FIQ for global fatigue within the resistance exercise group but there were no significant differences in change in the FIQ for global fatigue between the resistance exercise group and the control group. These results are in line with previous studies by Jones et al. [62] comparing resistance exercise with flexibility exercise, and by Häkkinen et al. [63] comparing resistance exercise with a control group in FM; both studies showed a significant change within the resistance exercise group in a VAS for global fatigue, but there were no significant differences in the change in global fatigue in comparison with controls. These findings endorse the use of a multidimensional assessment of fatigue in terms of physical fatigue, which was sensitive to change induced by resistance exercise.

Exploratory analyses were carried out in the present study aiming at investigating the effects of resistance exercise on the PSQI, the PCS and the HADS as compared to the active control group. In the analyses of change over time in the PSQI, the resistance exercise group had significant within-group improvements in the subscale for sleep quality and the active control group reported significant improvement in the subscale for needing medications to sleep, after the 15-week intervention. However, there were no significant between-group differences in change for any of the PSQI subscales. This is line with the results of a previous study of resistance exercise in women with FM [63].

There were significant within-group improvements over time in pain catastrophizing in the PCS total score and all three subscales in the resistance exercise group. However, there were no significant between-group differences in change, possibly because the control group also tended towards improvement in the PCS.

No significant changes were found for change in depression or anxiety assessed with the HADS in the resistance exercise group or the active control group. Also in previous exercise studies, the effect on depression and anxiety in FM has been found to be limited [31, 64].

Recent publications have recommended resistance exercise for patients with FM [65, 66]. The resistance exercise program in the present study had a person-centered approach and was progressively increased over 15 weeks. Over 60 % of the participants managed to increase the loads up to 80 % of 1 RM and the attendance was satisfactory at 71 %. Only 5 (7 %) of the participants in the resistance exercise group discontinued the intervention due to increased pain, which indicates that a majority of women with FM tolerate individually tailored resistance exercise twice a week for over 3 months. The resistance exercise program had a person-centered approach, which most likely contributed to the high attendance rate and low occurrence of adverse effects, as the approach enhances self-efficacy and sense of control in the participants. The resistance exercise program in the present study had similarities with the programs in the studies by Jones et al. [62] and Häkkinen et al. [63], which also were progressed and performed twice a week [62]. Relaxation therapy was chosen as the active control treatment in the present study and was assumed to improve overall wellbeing in the women with FM.

### Limitations

The present study is a sub-study and the statistical power was calculated with regards to the primary analysis published previously [32]. The number of included participants was also considered to be sufficient for the aim of the present study, and there was a significant difference between the groups in change in physical fatigue. The results of the present study must be interpreted with caution because the upper limit of the expected number of false significant results was calculated to be 0.21 for the primary outcomes, which indicates that 0–1 of the significant results could be false. A large number of tests of correlation were also performed in the analyses of predictors, and the values of explained variance (*R*^2^) in the multiple linear regression analyses were low, ranging from 0.05 to 0.14, which could be in the margin of error for the subscales.

Positive expectations of exercise have been found to play a role in the effect of exercise on psychological outcomes [67]. The participants in the present study were recruited by newspaper advertisement, which could have attracted persons with FM with expectations of improvement and a positive attitude towards resistance exercise. This might have influenced the magnitude of improvement and compliance with the exercise protocol. However, the same recruitment method was used for both groups and they were recruited simultaneously, thus the recruitment method would not have affected the outcome in group comparisons.

## Conclusions

The present study is the first to show that person-centered progressive resistance exercise contributed to improvement in physical fatigue in women with FM. Aspects of work and sleep were found to contribute to the improvement in fatigue.

## Abbreviations

ACR, American College of Rheumatology; CWP, chronic widespread pain; FIQ, Fibromyalgia Impact Questionnaire; FM, fibromyalgia; HADS, Hospital Anxiety and Depression Scale; LTPAI, Leisure Time Physical Activity Instrument; MFI-20, Multidimensional Fatigue Inventory; PCS, Pain Catastrophizing Scale; PSQI, Pittsburgh Sleep Quality Index; RCT, randomized controlled trial; RM, repetition maximum; VAS, visual analog scale
